# Silencing of one copy of the translation initiation factor eIFiso4G in Japanese plum (*Prunus salicina*) impacts susceptibility to *Plum pox virus* (PPV) and small RNA production

**DOI:** 10.1186/s12870-019-2047-9

**Published:** 2019-10-22

**Authors:** Julia Rubio, Evelyn Sánchez, David Tricon, Christian Montes, Jean-Philippe Eyquard, Aurélie Chague, Carlos Aguirre, Humberto Prieto, Véronique Decroocq

**Affiliations:** 1 Biotechnology Laboratory, La Platina Station, Instituto de Investigaciones Agropecuarias, Santa Rosa 11610, La Pintana, Santiago Chile; 2 Agronomical Sciences Doctoral Program, Campus Sur, University of Chile, Santa Rosa 11315, La Pintana, Santiago Chile; 3 Present address: Instituto de Ciencias Biomédicas, Universidad Autónoma de Chile, Providencia, Chile; 4 Present address: Integrative Genomics Doctoral Program, Universidad Mayor, Camino La Pirámide 575, Huechuraba, Santiago Chile; 5 INRA, UMR 1332 BFP, Equipe de virologie, 71 Avenue Edouard Bourlaux, 33883 Villenave d’Ornon, France; 6 Université de Bordeaux, UMR 1332 BFP, CS20032, 33883 Villenave d’Ornon, France; 7 Present address: Genetics and Genomics Doctoral Program, Iowa State University, 2437 Pammel Drive, Ames, IA 50011–1079 USA

**Keywords:** Plum, Sharka, Resistance, RNAi, Susceptibility gene, Translation initiation factor

## Abstract

**Background:**

In plants, host factors encoded by susceptibility (*S*) genes are indispensable for viral infection. Resistance is achieved through the impairment or the absence of those susceptibility factors. Many *S* genes have been cloned from model and crop species and a majority of them are coding for members of the eukaryotic translation initiation complex, mainly eIF4E, eIF4G and their isoforms. The aim of this study was to investigate the role of those translation initiation factors in susceptibility of stone fruit species to sharka, a viral disease due to *Plum pox virus* (PPV).

**Results:**

For this purpose, hairpin-inducing silencing constructs based on *Prunus persica* orthologs were used to generate *Prunus salicina* (Japanese plum) 4E and 4G silenced plants by *Agrobacterium tumefaciens*-mediated transformation and challenged with PPV. While down-regulated *eIFiso4E* transgenic Japanese plums were not regenerated in our conditions, *eIFiso4G11-*, but not the *eIFiso4G10-*, silenced plants displayed durable and stable resistance to PPV. We also investigated the alteration of the si- and mi-RNA profiles in transgenic and wild-type Japanese plums upon PPV infection and confirmed that the newly generated small interfering (si) RNAs, which are derived from the engineered inverted repeat construct, are the major contributor of resistance to sharka.

**Conclusions:**

Our results indicate that *S* gene function of the translation initiation complex isoform is conserved in *Prunus* species. We discuss the possibilities of using RNAi silencing or loss-of-function mutations of the different isoforms of proteins involved in this complex to breed for resistance to sharka in fruit trees.

## Background

Sharka, the most serious disease affecting stone fruit species (*Prunus* spp.), has socio-economic impact, especially on peach and plum orchards in Europe where the causal agent, the *Plum pox virus* (PPV), is endemic. PPV was classified as a quarantine pathogen, as one of the Top 10 Viruses in crops [1] and has cost 10 billion € over 30 years in Europe [2]. It affects crop species such as peach, plum, apricot, cherry and almond as well as ornamental and rootstock species [3]. Consequently, it requires significant effort to identify and deliver resistant germplasm which is, unfortunately, lacking in many stone fruit crop species, especially peach and Japanese plum [4].

In *Arabidopsis thaliana* (Arabidopsis), the eukaryotic translation Initiation Factor 4F complex (eIF4F) and its isoform (eIFiso4F) were reported to have an essential role in viral infection [5]. The eIF4F complex is composed of an mRNA cap-binding protein (eIF4E) and a large scaffold protein (eIF4G). Its counterpart, the eIFiso4F complex, is composed of the eIFiso4E and eIFiso4G isoforms. Because of their essential role in viral infection, the eIF4E and eIF4G translation initiation factors (and isoforms) are coded by host genes hereafter referred to as susceptibility (*S*) genes [6]. Consequently, mutations in one or the other of these *S* genes result in recessive resistance to viruses of the Potyviridae, Tombusviridae, Bromoviridae, Waikaviridae families. Indeed, *S* genes identified in Arabidopsis have been shown to be functionally conserved in crop species, including tomato, pepper, lettuce, barley, potato, tobacco, rice, pea [7–19]. We postulate here that it will also apply to the *Prunus* orthologues of the Arabidopsis *S* genes. A previous report described an essential role of the eIF (iso)4E host factor in infection of Arabidopsis by PPV [20]. Similarly, Nicaise et al [21] revealed that an Arabidopsis translationally non-functional *eifiso4g1* mutant is resistant to PPV, but not its *eifiso4g2* and *eif4g* knocked-out counterparts. While those previous studies demonstrated that the eIFiso4F complex is essential for PPV infection in Arabidopsis, genetic evidence obtained from other pathosystems indicated that potyviruses have a specific requirement for a given 4F or iso4F complex depending on the host plants. For example, *Lettuce mosaic virus* (LMV) uses eIFiso4E to infect Arabidopsis but requires eIF4E for lettuce infection [14]. Since in hexaploid European plums, eIFiso4E was shown to be involved in the resistance of *Prunus domestica* to PPV, it would indicate that in *Prunus spp.*, it is the eIF (iso)4F complex that is important for PPV infection [22]. However, eIFiso4E is in one single copy within the *Prunus* diploid genome [23] raising concerns of a possible fitness cost of virus resistance upon silencing of the *Prunus eIFiso4E*. This hypothesis could not be tested in the European plum *eIFiso4E*-silenced plants which unfortunately have died (Tian L et al, personal communication).

In this context, our research focused on identifying *S* genes for susceptibility to PPV in the diploid Japanese plum species, *Prunus salicina* that of stone fruit crop species is amenable to further genetic manipulation. We have demonstrated that in Japanese plum silencing the *eIFiso4G11* copy of the *Prunus* eIF4G isoform results in resistance to M and D PPV strains. However, while we confirm that PPV appears to recruit the host eIFiso4F complex for stone fruit tree infection, we were not able to regenerate viable *eIFiso4E*-silenced diploid plum trees. Our results suggest that translation initiation factors are functional susceptibility factors in *Prunus* and that silencing orthologs that have redundant copies results in stable and durable resistance to PPV with no adverse phenotypic effect on plant development.

## Results

**Fig. 1:**
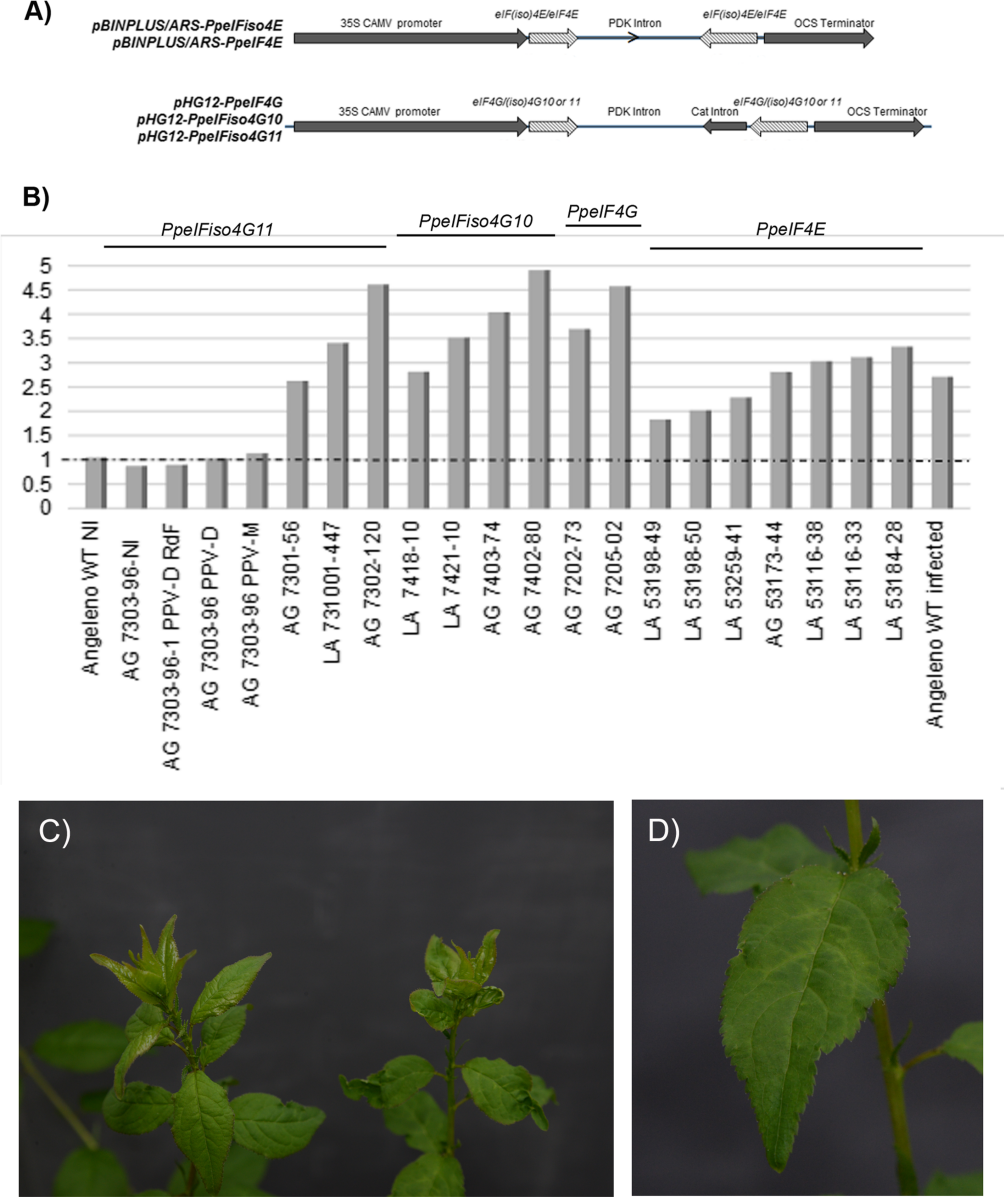
Production and sharka resistance of transgenic Japanese plum lines. **a** Schematic representation of the RNAi constructs used for Japanese plum transformation. PCR fragments of *PpeIF4E*, *PpeIFiso4E*, *PpeIF4G*, *PpeIFiso4G10*, and *PpeIFiso4G11* were cloned in opposite orientations as indicated by hatched arrows. **b** Assessment of resistance to PPV infection. Values represent the mean optical density values of three to four replicates per transgenic line tested for PPV infection over 3 to 5 vegetative cycles. Dotted line shows the basal OD value of the healthy negative control (‘Angeleno’). AG: ‘Angeleno’ transgenic lines; LA: ‘Larry Ann’ transgenic lines. Numbers starting with 73 were transformed with *pH 12-PpeIFiso4G11*; 74 with *pH 12-PpeIFiso4G10*; 72 with *pH 12-PpeIF4G* and 53 with *pBINPLUS/ARS-PpeIF4E*. All plants were grafted on rootstocks infected with PPV-M except NI (non-infected) and the plants noted PPV-D (PPV-D8 and PPV-D RdF Rouge de Fournés isolates). **c** Absence of symptoms on *PpeIFiso4G11-*silenced (left) and PPV symptoms on infected wild-type ‘Angeleno’ (right) Japanese plums. The susceptible plant on the right shows wilting of the bud leaves. **d** Close-up of PPV-infected ‘Angeleno’ leaf displaying chlorotic symptoms and vein clearing

Based on previous mapping studies performed in *Prunus armeniaca* (apricot), we identified full-length and functional *Prunus* orthologs for each *eIF* gene [23, 24]. Single loci encode proteins with a high level of amino acid identity with eIF4E (*Prupe.4G072600*), eIFiso4E (*Prupe.1G046600*) and eIF4G (*Prupe.2G118700*) translation initiation factors. In contrast, using BLAST analysis, we identified two distinct loci for eIFiso4G, *Prupe.1G395100* and *Prupe.7G265100* hereafter referred to as *PpeIFiso4G10* and *PpeIFiso4G11*, respectively [24]. In this report, we targeted the above five candidate loci for post-transcriptional gene silencing in order to assess the role of the *Prunus* eukaryotic initiation factors in susceptibility to PPV infection. For this purpose, we used a gene silencing strategy in which RNAi constructs were designed by cloning self-complementary hairpin structures either in the pBINPLUS/ARS or pHELLSGATE 12 vectors (Fig. 1a).

### Generation of transgenic Japanese plum trees

The RNAi constructs described in the Methods section were used to transform Japanese plum cultivars, Angeleno (AG) and Larry-Ann (LA), and seventy-three transgenic, *NPTII*-positive plants were obtained, of which seventeen were transferred to high confinement greenhouse at INRA (France) for PPV resistance testing (Additional file 7: Table S1). Noteworthy, although Wang et al [22] reported silencing of the Prunus *eIFiso4E* in hexaploid European plum, no diploid plum transformed with the *pBINPLUS/ARS-PpeIFiso4E* construct could be regenerated in our conditions. Thus this construct was removed from the rest of the study.

### One of the *pH 12-PpeIFiso4G11* transformed plants is resistant to PPV-M and PPV-D infection

Transgenic Japanese plum lines were initially inoculated with the PPV-M20 isolate (which belongs to the PPV-M strain) as described in Decroocq et al [25]. PPV-infected plants were maintained in high confinement greenhouse and scored for 3 to 5 vegetative cycles. One vegetative cycle comprises a 3-month period of cold followed by a 3-month period of growth during which we test twice by ELISA for PPV systemic infection of the 3 to 4 technical replicates of each Japanese plum transgenic line. Infection of the PPV susceptible rootstocks on which all transgenic lines and control plants were grafted was verified by symptom observation and serological tests. Once all rootstocks were confirmed PPV positive, viral systemic infection of the Japanese plum transgenic scions was scored by ELISA. Figure 1b summarizes the PPV resistance scoring data, over all vegetative cycles and technical replicates. After the first cycle of cold treatment, all non-transformed control plants (i.e. ‘Angeleno’ and ‘Larry Ann’) were PPV positive. Only one transgenic line, namely AG 7303–96, remained negative for PPV-M infection after five consecutive, vegetative cycles (Fig. 1b). Symptoms of PPV-M infection are displayed in Fig. 1c and d.

To test the durability of AG 7303–96 resistance, the same non-infected, transgenic line was multiplied by grafting on rootstocks inoculated either with PPV-D8 or PPV-D Rouge de Fournés. After three vegetative cycles, all replicates remained PPV-negative as depicted in Fig. 1b, thus attesting stable and durable resistance to PPV infection. Time point scoring of PPV infected transgenic lines, all along the three vegetative cycles, are displayed in Additional file 1: Figure S1.

### Reduction of the *PpeIFiso4G11* expression in transgenic Japanese plum plants is linked to resistance to PPV infection

**Fig. 2:**
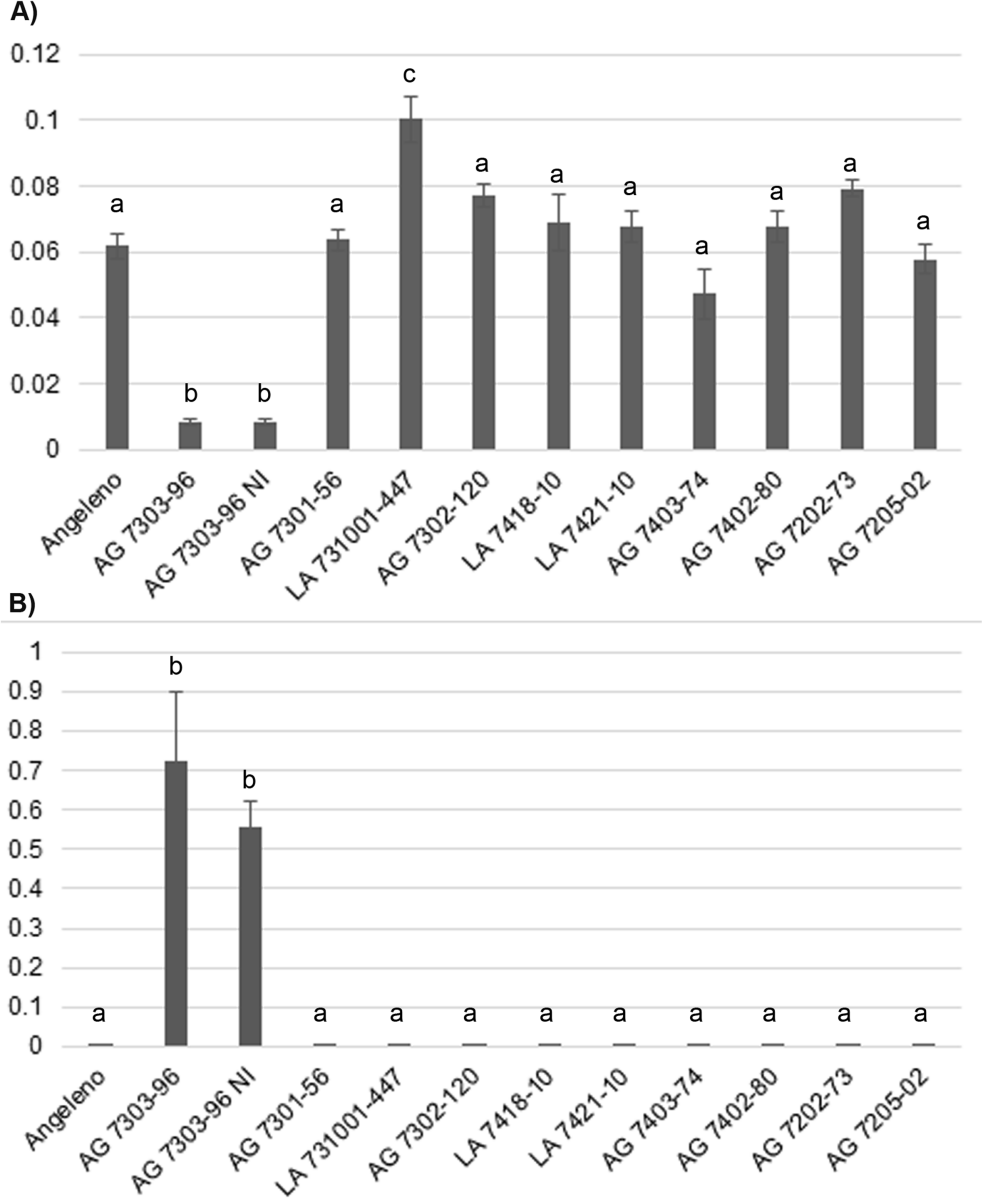
Relative expression analysis of *PpeIFiso4G11* and *PDK* intron in leaves of transgenic Japanese plum trees. Transcript levels were analyzed by qRT-PCR. All values were normalized to the *TEFII* reference gene and then compared to the wild type ‘Angeleno’ gene expression level. Error bars represent the standard deviation of two biological replicates each analyzed in triplicate. AG: transgenic ‘Angeleno’ plum lines; LA: transgenic ‘Larry Ann’ plum lines. All plants were grafted on rootstocks infected with PPV-M except NI (non-infected). The transgenic line numbers are corresponding to the ones depicted in Fig. 1b. The standard deviation between replicates is indicated by vertical lines. Statistical analysis was performed using the Kruskal–Wallis rank sum test in R software v. 3.2.5. Significantly different values are noted with lowercase letters (*P* value ≤0.05) when comparing the expression level between transgenic and wild-type lines. **a** qRT-PCR estimation of the *PpeIFiso4G11* transcript levels in transgenic Japanese plum lines. **b** Transcriptional expression of the PDK intron in transgenic Japanese plum lines. Note that only the pH 12-PpeIF4G, pH 12-PpeIFiso4G10 and pH 12-PpeIFiso4G11 transformed lines were tested here

The effectiveness and specificity of the silencing induced by the RNAi constructs were determined by estimating the relative expression of the targeted genes coding for eukaryotic initiation factors in each transgenic Japanese line (Fig. 2 and Additional file 2: Figure S2). While the AG 7303–96 transgenic line displays a significant reduction in *PpeIFiso4G11* transcript levels (Fig. 2), none of the other target genes appears to be silenced (Additional file 2: Figure S2A to C). The same applies for the over-expression of the *PDK* intron in the AG 7303–96 plants but not in the other transgenic lines (Fig. 2). This raises the hypothesis that all the other transgenic clones depicted in Additional file 7: Table S1 were chimeras, being *nptII*-positive during the micropropagation but not later, after grafting. Interestingly, the *PpeIFiso4G10* transcript level is also partly reduced (but not totally) in the AG 7303–96 Japanese plum plants (Additional file 2: Figure S2A).

### The production of 21- and 24-nucleotides (nt) long molecules targeting the MIF central domain of *PpeIFiso4G11* is linked to PPV resistance

**Fig. 3:**
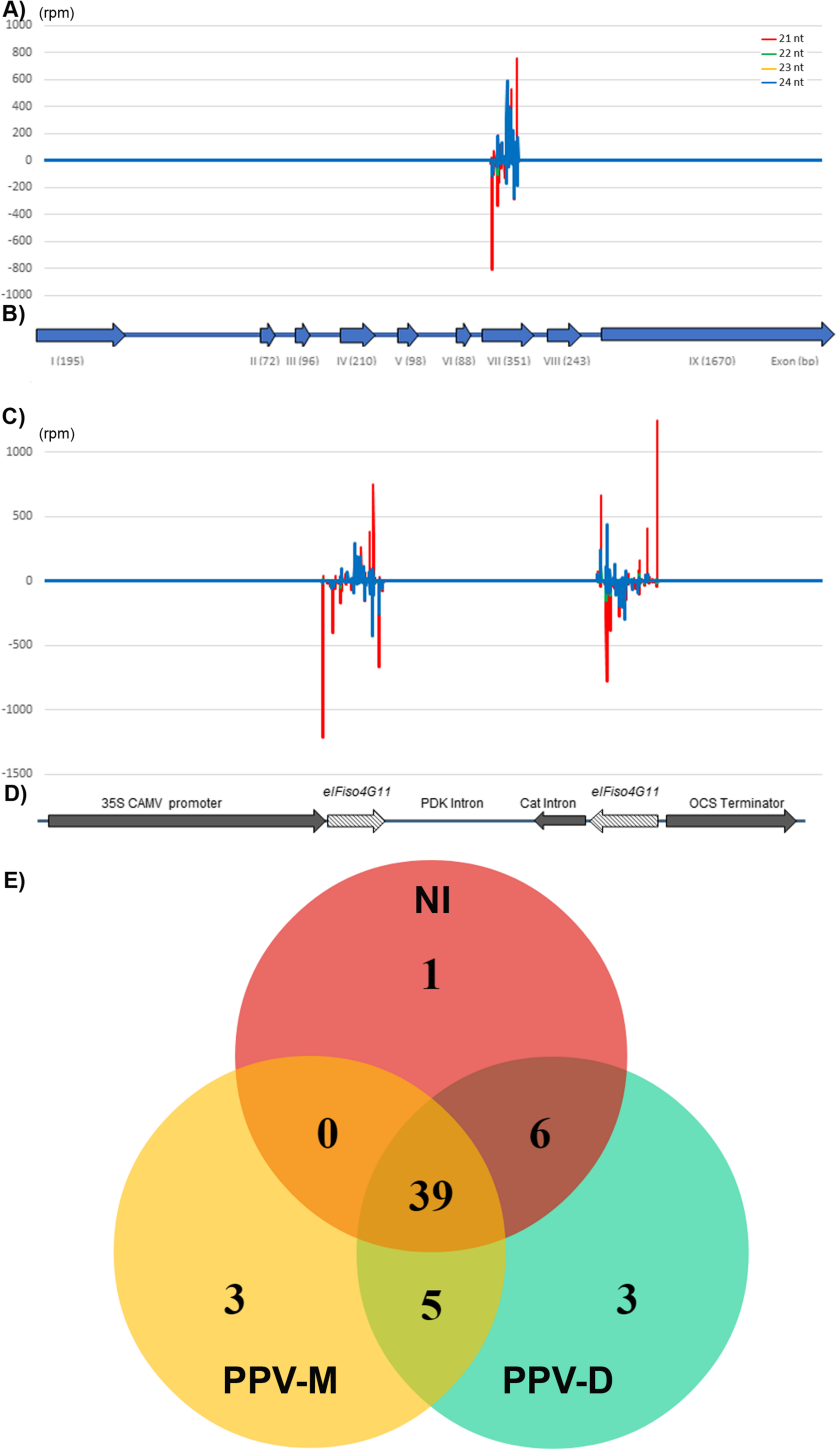
Si- and miRNA patterns in *P. salicina PpeIFiso4G11-*silenced plants. **a** Accumulation of *PpeIFiso4G11-MIF* specific si-RNA in the AG7303–96 leaves. (rpm) reads per million with a total count of 289,133 reads over the *Prupe.7G265100* locus. **b** Schematic representation of the *Prupe.7G265100* gene sequence coding for the Prunus eIFiso4G11 factor. Roman numbers depicted under the sequence indicate exons with (I) being the first exon that contains the start codon and VII corresponds to the MIF4G domain. **c** Mapping of *PpeIFiso4G11-MIF* specific si-RNA reads over the *pH 12-PpeIFiso4G11* construct (**d**). **e** Venn diagram for the specific and shared miRNAs among the non-infected (NI), PPV-M or PPV-D infected AG7303–96 libraries

To gain a better understanding of the susceptibility gene silencing defense process, the expression profiles of siRNAs and miRNAs with and without viral infection in both wild type and transgenic Japanese plums were examined by small RNA high-throughput sequencing. We show here that the AG 7303–96 transgenic line constitutively produces siRNA molecules between 21- and 24-nucleotides (nt) long mapping over the VII exon of the *PpeIFiso4G11* (Prupe.7G265100) gene that corresponds to the initially targeted MIF4G domain (Fig. 3a-d). The number of siRNA mapping molecules (in total 114,601 reads per million) is 3400 and 14,456 times higher than molecules that scattered all over the *PpeIFiso4G10* and *PpeIF4G* loci, respectively (Additional file 3: Figure S3). The production of *PpeIFiso4G11*-specific siRNA was also double checked by stem-loop reverse transcription-polymerase chain reaction (RT-PCR) (Additional file 8: Table S2 and Additional file 4: Figure S4A).

### Pattern of miRNA production in PPV resistant and susceptible Japanese plum trees

The above small RNA NGS data was used to analyze the contribution of miRNAs in response to viral infection. We examined the expression profiles of miRNAs upon viral or mock infection grafting in both wild type and transgenic Japanese plums (Additional file 9: Table S3A-C, Fig. 3e, Additional file 5: Figure S5). We also verified few of the small RNA sequencing data by stem-loop RT-PCR (Additional file 8: Table S2). Interestingly, we retrieved a common marker for viral infection, miR171, that was already shown to accumulate in *Nicotiana benthamiana* infected with PPV [26], in rice infected with RSV (Rice Stripe Virus, [27]) and in *Nicotiana tabacum* infected with PVX (Potato Virus X, [28]). In our case, miR171 accumulates only in PPV-infected conditions, both in wild type ‘Angeleno’ and in *PpeIFiso4G11*-silenced AG 7303–96 transgenic line. The induction of miR171 expression in the resistant transgenic line is intriguing, however we cannot rule out the possibility of a translocation of the miR171 small molecules from PPV-infected rootstock to the scion or the limited but still existing movement of viral particles within the resistant scion that could trigger miR171 induction. This hypothesis was tested by stem-loop RT-PCR (Additional file 4: Figure S4B), in which we detected miR171 in both scion and rootstock samples infected with PPV-M, only.

Two other small RNAs of interest are miR399 and miR168, which accumulate only in wild type ‘Angeleno’ inoculated with either PPV-M or PPV-D and were never detected in healthy plants or in PPV-resistant AG 7303–96 transgenic line, infected or not (Additional file 8: Table S2, Additional file 9: Table S3A and B).

### Pattern of miRNA production in non-infected, wild type and transgenic *PpeIFiso4G11*-silenced, Japanese plum trees

Among the small RNA molecules which are uniquely expressed either in wild type ‘Angeleno’ or in the *PpeIFiso4G11*-silenced transgenic line (Additional file 9: Table S3C and Additional file 6: Figure S6), two miRNAs are worth noting: miR156 and miR172. Both miRNAs are involved in the juvenile-to-adult transition from the juvenile to the adult phase of plant development, through a sequential action: miR156 acts as a master regulator of the vegetative phase by repressing downstream transcription factors including miR172, which promotes flowering when over-expressed [29]. In our study, miR156 accumulates in the AG 7303–96 transgenic line, while miR172 accumulates in non-transgenic Japanese plum, in the absence of miR156 (Additional file 9: Table S3C). Stem-loop RT-PCR detection of miR156 and miR172 in wild type ‘Angeleno’ and AG 7303–96 transgenic line only partially confirmed small RNA high-throughput sequencing. Indeed, in this validation step, miR172 accumulates in both transgenic and non-transformed ‘Angeleno’ plants (Additional file 8: Table S2). However, this means that, while a normal vegetative growth is currently observed among the AG7303–96 plants, we will pay attention to the juvenility period and flowering ability of the *PpeIFiso4G11*-silenced transgenic line.

## Discussion

**Fig. 4:**
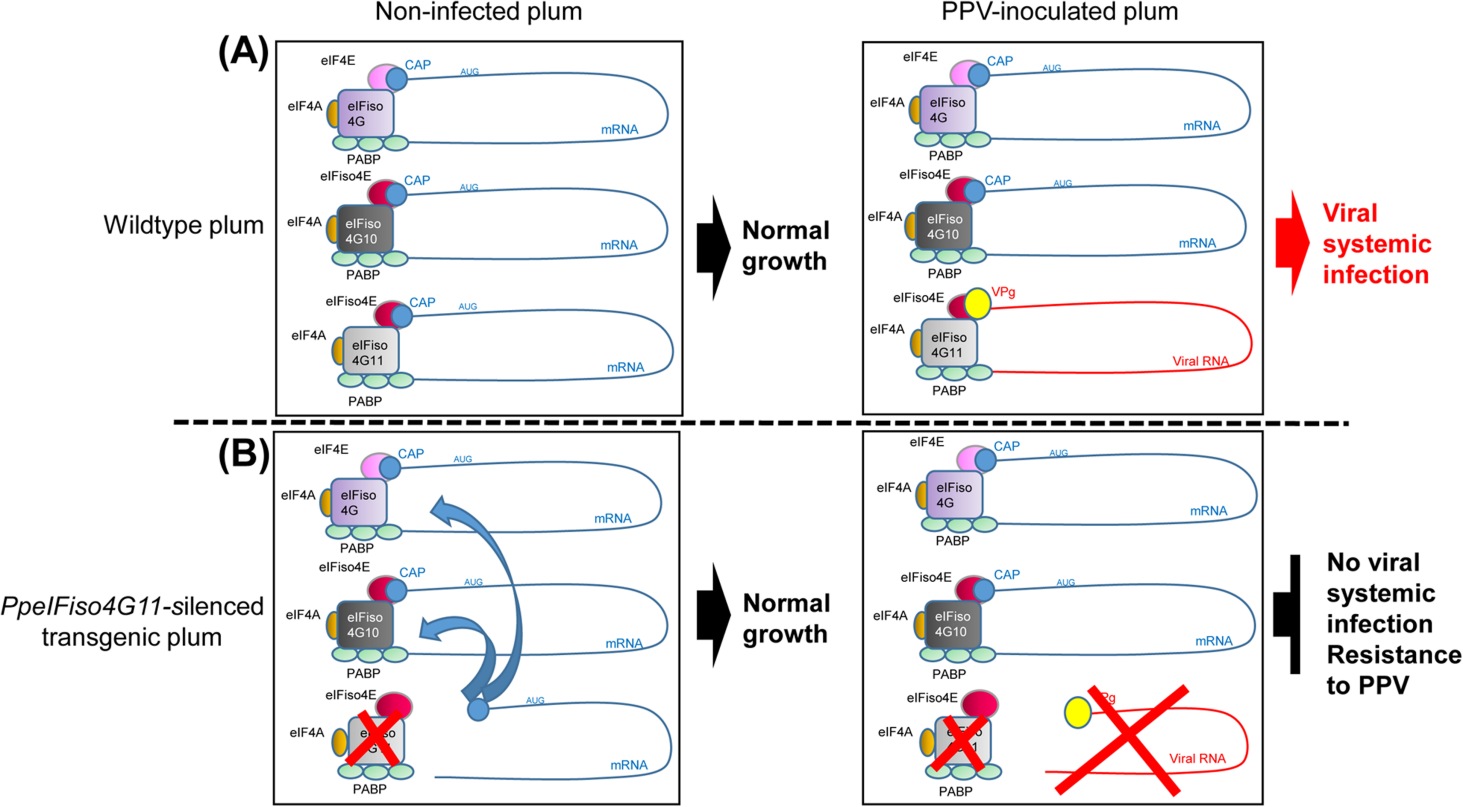
Model of eIFiso4F-mediated susceptibility to PPV in diploid plum. **a** In diploid *Prunus* species, the eIF4F translation initiation complex is composed of eIF4E which interacts with the mRNA (m7Gppp) cap, eIF4G which interacts with both eIF4E and the polyadenosine-bound PolyA binding proteins (PABP), and eIF4A. On the other hand, two copies of eIFiso4G (*PpeIFiso4G10* and *PpeIFiso4G11*) are transcribed and the corresponding proteins are both able to form the eIFiso4F complex, in interaction with a single PpeIFiso4E isoform, PABP and eIF4A (A, left panel). In this model, the only eIF4G and isoform used by PPV is PpeIFiso4G11. In non-transgenic, wild type plum infected with PPV, interaction of the viral genome-linked protein, VPg, with the eIFiso4F complex that involves PpeIFiso4G11 is leading to plant susceptibility (A, right panel, in red). The viral RNA genome is represented in red and the host mRNA, in blue. **b** In *PpeIFiso4G11*-silenced plum, host cell messenger RNAs are still recruiting the eIF4F or/and eIFiso4F complex that involves *PpeIF4G* and *PpeIFiso4G10*, respectively (B, left panel), thus preventing abnormal growth of the plant. However, the virus is no longer able to hijack the eIFiso4F complex because of the absence of PpeIFiso4G11 factor (B, right panel). It results in a failing viral cycle, the virus being impaired either in its translation, replication and/or cell-to-cell movement

This study establishes a new strategy for durable and stable resistance to sharka in stone fruit trees. We show that the down-regulation of one of the Prunus translation initiation factor *eIFiso4G* genes is linked to the production 21- to 24-nt long *PpeIFiso4G11* specific siRNAs. Downregulation of *PpeIFiso4G11* expression is here sufficient to contain virus infection over at least five consecutive vegetative periods and for at least two different viral strains, PPV-M and PPV-D (see the model proposed in Fig. 4). Similarly, Rodríguez-Hernández et al [30] showed that the efficient silencing of the *Cucumis melo* eukaryotic translation Initiation Factor 4E gene is correlated with the appearance of 21- to 24-nt *Cm-eIF4E*-derived small interfering RNAs (siRNAs), thus leading to resistance to four distinct viruses. In our case, it confirms previous results obtained with the Arabidopsis or *P. domestica*/PPV pathosystems from which it was hypothesized that PPV requires a functional eIFiso4E/eIFiso4G complex (e.g. eIFiso4F) to infect its host but not its eIF4F counterpart [20–22]. In spite of some functional redundancy in host protein translation for those cognate complexes, potyviruses demonstrate notable isoform specificity, with eIF4F and eIFiso4F complexes usually having distinct and non-overlapping functions in virus infection [31]. In consequence, we propose a model of eIFiso4F hijacking by PPV in stone fruit tree, as depicted in Fig. 4.

In Arabidopsis, no adverse effects on plant growth were observed among *eif4e* or *eifiso4E* loss-of-function mutants, except in the double mutants [32, 33] indicating a functional redundancy between the eIF4E forms. However, in Arabidopsis, *eIF4E* is present in three copies, i.e. At4g18040, At1g29690 and At1g29550 whereas *eIFiso4E* maps to one single locus, At5g35620 [5]. In comparison, in peach, only one locus corresponds to each isoform of the *eif4E* gene (*Prupe.4G072600* for *PpeIF4E* and *Prupe.1G046600* for *PpeIFiso4E*), thus limiting a possible complementation between the isoforms. This could explain our inability to regenerate *eifiso4E*-silenced plants since *P. salicina* is diploid. On the contrary, Wang et al [22] were able to test *eifiso4E*-silenced European plum lines, possibly because of the higher ploidy level (hexaploid) of this host.

Among the crop species, another case of resistance to viruses linked to the eIF4G factor or its isoform was documented in rice. Resistance to RYMV (*Rice Yellow Mottle Virus*, a Sobemovirus) is correlated with mutations in *eIFiso4G* [7] while RTSV (*Rice Tungro Spherical Virus*, a Sequiviridae) is recruiting the eIF4G translation initiation factor [12]. Interestingly, *eIF4G* alleles resulting in a truncated form could not be obtained in homozygous state, demonstrating that a functional eIF4G is essential for normal growth in rice [34].

In the current study, we also examined the production of small noncoding RNA molecules such as miRNAs and siRNAs in both wildtype and transgenic plums and their contribution in response to viral infection. Indeed, many studies in the past decade showed that plant viruses alter small RNA profiles, thus impacting host gene regulation (for review see [35, 36]). Upon PPV-infection, we identified small miRNAs already associated with viral infection among which, miR171 [26]. This small RNA was detected in PPV-resistant (*PpeIFiso4G11*-silenced) or susceptible (wildtype) scions grafted on PPV-infected rootstocks. This result suggests that miR171 is not linked to susceptibility/resistance to sharka but is instead a marker of PPV infection that can be transferred from infected, susceptible rootstock to PPV-resistant scion.

We also identified two other small RNA which were previously related to viral infection, miR399 and miR168 namely. Similarly, miR399 was previously described to be highly expressed upon RSV infection of wild type non-transgenic rice plants but not in RSV-resistant transgenic lines, engineered with the virus-derived ihpRNA strategy [37]. A similar situation is observed for miR168, a regulator of AGO1 mRNA, which displays enhanced accumulation in wild type Japanese plum infected with either PPV strains (this study) but also in Arabidopsis or *N. benthamiana* inoculated with CymRSV (*Cymbidum ringspot virus*) [38]. Therefore, those two small RNAs appear to be associated with host susceptibility to viral infection.

Albeit we showed that the *PpeIFiso4G11*-derived siRNAs are the trigger for resistance to PPV, the effect of the mitigation of *PpeIFiso4G11* expression on Japanese plum development was also questioned. No pleiotropic and visible effect on plant developmental features was observed for the *PpeIFiso4G11*-silenced plants, up to now, but care will be taken concerning the juvenility period and flowering ability of the AG 7303–96 transgenic line.

## Conclusion

We demonstrated in the current study that, although one single copy of *PpeIFiso4G* (i.e. *PpeIFiso4G10*) is sufficient to warrant normal growth of transgenic, diploid plum, a functional copy of *PpeIFiso4G11* is indispensable for PPV infection (Fig. 4). Therefore, the strategy of knocking out a host gene such as *PpeIFiso4G11*, or at least impairing the plum/PPV interactions through the selection of non-functional PpeIFiso4G11 protein could be an alternative approach to Pathogen-Derived Resistance (PDR) as described by [39]. When natural resistance is not available and non-functional alleles are lethal, the new CRISPR/Cas9 genome editing system would be another promising strategy, allowing targeted mutations in low-copy, susceptibility genes in plants.

## Methods

### Plant materials

Seed explants ‘Angeleno’ and ‘Larry Ann’ for plum gene transfer and regeneration experiments were obtained from certified commercial orchards (Fundo Quilamuta, Verfrut Rapel, Rapel Valley, Metropolitan Region, Chile). Peach ‘GF305’ plants used for grafting and nucleic acids extraction were produced by the Lafond nurseries (Valréas, France).

### Identification of orthologous eIF4G and isoform genes from *Prunus persica*

Sequence information for the Arabidopsis *eIF* genes was obtained from the TAIR (*Arabidopsis thaliana* genome v10, http://www.arabidopsis.org) database and used in comparative searches for the putative *Prunus persica* orthologues of the *eIF4G* (*PpeIF4G*) and *eIFiso4G* (*PpeIFiso4G*) genes using BLAST in the Phytozome v10.1 (*Prunus persica* genome v2.1, http://www.phytozome.net) database [40]. Different copies of the *eIFiso4G* orthologue were obtained and named *eIFiso4G10* and *eIFiso4G11* based on a previous study [24] (see GenBank accessions EU558279 and EU558280, respectively). Additional sequence alignment was performed using the ClustalW software [41].

### Design and cloning of Prunus-silencing constructs

Silencing fragments were defined for each *P. persica* orthologous gene based on the parameters described by the RNAiweb platform (http://www.rnaiweb.com/RNAi/siRNA_Design/) [42]. The stability of the hairpin structure and the number of possible sequences generated from each hairpin were predicted using OligoWalk (http://rna.urmc.rochester.edu/servers/oligowalk.html) [43].

#### Peach total RNA extraction

One hundred milligrams of ‘GF305’ leaves were collected from in vitro peach plantlets, frozen in liquid nitrogen, ground and mixed with the extraction buffer from the InviTrap Spin Plant RNA Mini Kit (Thistle Scientific, Ltd., Glasgow, UK). The remaining procedures were carried out using this kit according to the instructions provided by the manufacturer.

#### Complementary DNA (cDNA) synthesis and silencing arm cloning and sequencing

Primers complementary to the target sequences were designed to amplify ±200 bp long fragments (Additional file 10: Table S4). To limit simultaneous silencing of the isoforms (eIF4E vs eIFiso4E or eIF4G vs eIFiso4G) or/and of the paralogs (eIFiso4G10 vs eIFiso4G11), the most divergent sequences were selected to design the gene silencing constructs. Restriction sites were added to the primer sequences to facilitate subcloning of the target fragments (see Additional file 10: Table S4 for details).

One microgram of total RNA was treated with DNase I (Thermo Fisher Scientific, Waltham, Massachusetts, USA) to eliminate genomic DNA contamination. For cDNA synthesis, the Superscript II RT system (Thermo Fisher Scientific) was used according to the manufacturer’s instructions. Two hundred nanograms of cDNA were mixed with 1.25 U of High Fidelity PCR Enzyme Mix (Thermo Fisher Scientific), 5 μl of 10X High Fidelity buffer, 0.4 mM of dNTPs mix, and 0.5 μM of each primer (Additional file 10: Table S4), and the final volume was adjusted to 50 μl. Amplifications were performed using an Eco System (Illumina, San Diego, California, USA) according to the following thermal profile: 1 min at 94 °C; 35 cycles of denaturation at 94 °C for 20 s, annealing at 55 °C for 20 s, and extension at 72 °C for 30 s; and a final extension of 2 min at 72 °C. The amplified fragments were cloned into the pGEM-T Easy vector (Promega, Madison, Wisconsin, USA), confirmed by sequencing (Macrogen, Seoul, Korea) and identified as *P. persica* (Pp) *eIF4E* (*PpeIF4E*), *PpeIFiso4E*, *PpeIF4G*, *PpeIFiso4G10*, and *PpeIFiso4G11*.

#### Silencing constructs

Following PCR amplification of the target fragments, *PpeIF4E* and *PpeIFiso4E* were subcloned into the pHannibal vector on both sides of the pdk intron [44], then the intron-containing construct(s) were transferred into the Not1 restriction site of pSPORT2 plasmid (Addgene) before a final *Eco*R1*-Hin*dIII transfer into the pBINPLUS/ARS binary vector [45]. In the meantime, *PpeIF4G*, *PpeIFiso4G10*, and *PpeIFiso4G11* silencing arms were recombined into the pENTR/D entry vector (Thermo Fisher Scientific). Subsequently, 150 ng of entry vector were incubated with 150 ng of the vector pHellsgate12 [46] in the presence of the LR Clonase II enzyme mix (Thermo Fisher Scientific) according to the manufacturer’s instructions. The constructs (noted pBINPLUS/ARS- or pHG12- depending on the receiving binary vector) were confirmed by sequencing (Macrogen) and identified as pBINPLUS/ARS-PpeIF4E, pBINPLUS/ARS-PpeIFiso4E, pHG12-PpeIF4G, pHG12-PpeIF (iso)4G10, and pHG12-PpeIF (iso)4G11. Each resulting construct contains the target eIF gene fragment in forward and reverse orientations separated by a single pdk intron for pBINPLUS/ARS [45] and a double pdk and cat intron for pHellsgate12 [46].

### *Prunus salicina* genetic transformation and regeneration

Each pBINPLUS/ARS- or pH 12- construct was used to transform the *Rhizobium radiobacter* (Sinom. *Agrobacterium tumefaciens*) GV3101 strain. Competent Agrobacterium cells were electroporated following Urtubia et al. [47], using a Gene Pulser (Bio-Rad, California, USA) and the following conditions: 1.25 V, 400 Ω, and 25 μF. The resulting Agrobacterium clones containing each construct (Agrobacterium pBINPLUS/ARS- or pHG12- clones) were used for the genetic transformation of hypocotyl medial segments of ‘Angeleno’ and ‘Larry Ann’ mature seeds [47]. Buds from segments were induced to regenerate following the procedures indicated by these authors, although without the use of a selection agent. No selection conditions were kept for 2 months until individual plant formation was evident.

### Selection and characterization of Japanese plum transgenic lines

Plants were subjected to selection using the same regeneration medium by increasing kanamycin concentrations every 2 weeks (25, 50, 75, 100 mg/L). After 2 months in kanamycin 100 mg/L, resistant lines were subjected to primary characterization by PCR. Genomic DNA (gDNA) was prepared from leaf samples from T0 plants as described in [48] using a modified extraction buffer [cetyl trimethylammonium bromide (CTAB) 2% (p/v), Polyvinylpyrrolidone (PVP) 40,000 2% (p/v)), Tris-HCl (pH 8.0) 100 mM, EDTA 25 mM, NaCl 2 M, spermidine 0.05% (p/v), ß-mercaptoethanol 2%]. Quantification of gDNA was carried out using the Bio Spec-nano computer (Shimadzu, California, USA). Fifty nanograms of gDNA were used in amplification reactions; as a control reaction, the *Prunus* Translation Elongation Factor 2 (TEF2) (Prupe.4G138700) was amplified using primers TEF2_F1 and TEF2_R1, resulting in a 129 bp long fragment. For the *npt*II gene (Genebank: AJ311874.1), the primer pairs NPTII_F5/NPTII_R5 or NPTII_int_Fw/NPTII_int_Rv were alternatively used, giving rise to a fragment of 687 and 399 bp respectively. For the *Rhizobium radiobacter*, syn. *Agrobacterium radiobacter* virG gene (GenBank: NG_034482.1), the primers virG_Fw and virG_Rv generated a fragment of 391 bp. The amplification reactions were performed according to previously described conditions [47]. Primers are presented in the Additional file 10: Table S4.

### *Plum pox virus* (PPV) resistance phenotyping of the Japanese plum transgenic lines

Three to four replicates per transgenic line were challenged with PPV for three to five consecutive vegetative cycles (see protocol for PPV resistance testing in [25]) and compared to similar groups of control, non-inoculated, plants (‘Angeleno’ and ‘Larry-Ann’). A PPV-M isolate (namely PPV-M20), highly virulent on apricots [25], was used in a first round of PPV resistance screening. PPV-M resistant lines were then tested with isolates belonging to the PPV-D strain (PPV-D8 and PPV-D Rouge de Fournés). Systemic infection by PPV was verified by ELISA, twice per vegetative cycle. Optical density data was normalized as described in [25]. The mean OD value was then averaged over the three to five consecutive vegetative cycles and finally standardized over the negative control used throughout the PPV resistance scoring (non-infected, ‘Angeleno’ mean OD value).

### Small RNA massive sequencing

Massive sRNA sequencing was carried out using PPV-M or PPV-D infected, resistant or susceptible transgenic plants. Non-transformed (hereafter called wild type) ‘Angeleno’ plants were added to the analysis. All leaf samples were collected in the third vegetative cycle of PPV testing. Small RNA was extracted following the protocol described in [49]. Libraries and sequencing procedures are detailed by [49] using a MiSeq (Illumina) sequencer.

### Si- and miRNA identification and target prediction

Small interfering RNA (siRNA) reads, obtained from non-transformed (wild type) and transgenic *Prunus salicina* ‘Angeleno’ small RNA libraries upon mock and viral infection conditions, were analyzed using the CLC Genomics Workbench software (CLC Bio, Aarhus, Denmark) as indicated in [49]. Sequences between 21- and 24-nt long were selected and then aligned against the *P. persica eIF4G* (Prupe.2G118700.1) and *eIFiso4G* coding loci (Prupe.7G265100.1 for *PpeIFiso4G11* and Prupe.1G395100.1 for *PpeIFiso4G10*). High penalty settings for filtered reads annealing to template sequences were established as in Montes et al. [49]. Further analysis was performed using Microsoft Excel 2013 (Microsoft, Redmond, WA, USA) and home-made scripts for plotting.

Prunus miRNA species were obtained from the previously filtered small RNA datasets by mapping the reads using CLC (CLC Bio) to the miRbase dataset [50]. Mapped miRNAs from each experiment (Angeleno non-infected, Angeleno+PPV-M, Angeleno+PPV-D, *PpeIFiso4G11*-silenced Angeleno non-infected, *PpeIFiso4G11*-silenced Angeleno+PPV-M, *PpeIFiso4G11*-silenced Angeleno+PPV-D) were normalized and molecules showing a cut-off > 1 read per million were selected. Further comparative analyses among miRNA datasets were performed using Microsoft Excel 2013 (Microsoft) and home-made scripts (available under request). Candidate target genes were obtained by submitting the selected molecules to the psRNA Target server - 2017 release [51]. Target genes showing the best expectation number for each miRNA as described in [51] were deduced using the peach reference genome [52].

### RT-qPCR analysis to determine *eIF4E*, *eIF4G* and isoforms mRNA expression levels in Japanese plum transgenic plants

Total RNA was extracted from leaves of PPV-challenged transgenic lines collected in the third cycle of PPV testing. They were treated with Turbo DNaseI (Thermo Fisher Scientific) before complementary DNA synthesis with the Superscript II® reverse transcriptase from Invitrogen/ Revertaid/Ribolock reverse transcriptase kit (Fermentas), using an oligo-dT (16) primer. Relative qPCR to quantify *PpeIF4E*, *PpeIF4G*, *PpeIFiso4G10* and *PpeIFiso4G11* mRNA accumulation was performed on a Light Cycler 480 II machine (Roche Diagnostics) by using LightCycler® 480 SYBR Green I master and one tenth of the newly synthesized cDNAs. Expression of *TEFII* (Prupe.4G138700) was used as internal reference. Based on re-sequenced Japanese plum copies, specific primers were designed for each copy of the *Prunus* translation initiation factor genes as well as for the internal reference gene (Additional file 10: Table S4). RT-qPCR procedures for cycling conditions and relative expression statistical analysis are detailed elsewhere [53]. Values statistically different when comparing the expression level of wild-type and transgenic lines were verified by analysis of summary rank with the Kruskal-Wallis test with the R v 3.2.5 software.

### Stem-loop reverse transcription-polymerase chain reaction (RT-PCR) detection of small non-coding RNAs

End-point looped RT-PCR was used to detect the target small RNA species derived from NGS analyses. Stem-loop primers were designed for both miRNAs and siRNA according to Castro et al. [54], using the “Stem-loop primer designer” option from the “amiRNA designer” tool available in the web page www.fruit-tree.genomics.com, tab “Biotools”. Primer sequences are presented in Additional file 8: Table S2. For reactions, total RNAs from leaf samples in the 5th vegetative cycle were processed as indicated by Sánchez et al. [55]. Five hundred nanograms of total RNA were mixed with 1 μL of each Loop primer (stock 10 mM) and 0.5 μL of each dNTP (10 mM) in a final reaction volume of 15 μL. The mixture was incubated for 5 min at 65 °C and then chilled on ice for 2 min. Four microliters of 5X First Strand buffer (Thermo Fischer Scientific) were mixed with 2 μL of 0.1 M DTT, 0.1 μL of RNase-OUT (40 U/μL) (Invitrogen, USA) and 0.25 μL of Superscript II reverse transcriptase (200 U/μl) (Thermo Fischer Scientific). The mixture was spun down, and “pulsed RT” was applied using an Eppendorf Mastercycler Nexus thermal cycler (Thermo Fisher Scientific) set as follows: 30 min at 16 °C and 60 cycles of 30 s at 30 °C, 30 s at 42 °C and 1 s at 50 °C. The mixture was incubated for 5 min at 85 °C. The amplified product was used as a template for a second PCR in which the Reverse primer was mixed with the corresponding Forward primer (Additional file 8: Table S2). The PCR conditions were 3 min at 95 °C and 35 cycles of 30 s at 95 °C, 30s at 60 °C and 30 s at 72 °C. A final extension was applied for 5 min at 72 °C. The PCR products were separated by agarose gel electrophoresis using 3% Low Range agarose (Bio-Rad) with a 25 bp Molecular Weight Standard (Thermo Fisher Scientific) and alternately using a Fragment Analyzer automated parallel capillary electrophoresis system.

Small interfering RNAs from the hairpin showing the highest reads per million in the NGS experimentation were selected for these PCR detections. Nomenclature for these molecules was based on their corresponding number of reads (Additional file 8: Table S2).

## Supplementary information

**Additional file 1: Figure S1.** DAS-ELISA of wild type and transgenic Japanese plum lines following inoculation with *Plum Pox Virus* (PPV) over three vegetative cycles. Values represent the mean optical density values of three to four replicates per transgenic line tested for PPV infection over 3 vegetative cycles. AG: ‘Angeleno’ transgenic lines; LA: ‘Larry Ann’ transgenic lines. Numbers starting with 73 were transformed with *pH 12-PpeIFiso4G11*; 74 with *pH 12-PpeIFiso4G10* and 72 with *pH 12-PpeIF4G*. All plants were grafted on rootstocks infected with PPV-M except NI (non-infected) and the plants noted PPV-D (PPV-D8 and PPV-D RdF Rouge de Fournés isolates).

**Additional file 2: Figure S2.** Relative expression analysis of *PpeIFiso4G10* (A), *PpeIF4G* (B) and *PpeIF4E* (C) in leaves of transgenic Japanese plum trees. Transcript levels were analyzed by qRT-PCR. All values were normalized to the TEFII reference gene and then compared to the wild type ‘Angeleno’ gene expression level. Error bars represent the standard deviation of two biological replicates each analyzed in triplicate. The standard deviation between replicates is indicated by vertical lines. Statistical analysis was performed using the Kruskal–Wallis rank sum test in R software v. 3.2.5. Transgenic and wild type Japanese plum lines labelled with the same letter are statistically identical (*P* value < 0.05). No significantly different values of *PpeIF4G* (B) and *PpeIF4E* (C) expression was evidenced by the Kruskal-Wallis test at *P* value ≤0.05. AG: transgenic ‘Angeleno’ plum lines; LA: transgenic ‘Larry Ann’ plum lines. The transgenic line numbers are corresponding to the ones depicted in Fig. 1b.

**Additional file 3: Figure S3.** Accumulation of *PpeIFiso4G10* (A) and *PpeIF4G* (C) specific siRNA in the AG7303–96 transgenic line. (B) is representing *PpeIFiso4G10* and (D) *PpeIF4G* sequences. (rpm) reads per million with a total count of 85 and 20 reads over the *PpeIFiso4G10* and *PpeIF4G* loci, respectively.

**Additional file 4: Figure S4.** Stem-loop reverse transcription-polymerase chain reaction (RT-PCR) detection of selected siRNAs and miRNAs. (A) Transgene-derived siRNAs (#6261 and 7361, respectively) were detected in AG 7303–96 transgenic Japanese plums as well as in peach GF305 rootstock (for #7361 exclusively). (B) Expression pattern of miR171e 3p in scions and rootstocks of non-transformed ‘Angeleno’ and transgenic AG 7303–96 Japanese plum trees.

**Additional file 5: Figure S5.** Venn diagram for the specific and shared miRNAs among the wild type, non-transgenic and AG7303–96 transgenic ‘Angeleno’ libraries.

**Additional file 6: Figure S6.** Venn diagram for the specific and shared miRNAs among the non-infected (NI), PPV-M or PPV-D infected wild type (non-transgenic) ‘Angeleno’ libraries.

**Additional file 7: Table S1.** Summary of Japanese plum transformation and resistance testing. ^1^ Number of *Prunus salicina* explants treated with *Agrobacterium tumefaciens*. The constructs are corresponding to the ones depicted in Fig. 1a, except *pBINPLUS/ARS-PpeiFiso4E* for which we could not regenerate viable, transgenic clones.

**Additional file 8: Table S2.** Stem-loop reverse transcription-polymerase chain reaction (RT-PCR) detection of selected siRNAs and miRNAs. * Number indicates the number of reads issued from NGS for each siRNA molecule as depicted in Fig. 3. Loop primer: lower case fonts indicate complementary residues in the loop; bold, italic upper case indicate hybridizing sites for the reverse primer; double underlined fonts, residues hybridizing with the small RNA. Forward primer: double underlined fonts indicate residues hybridizing with the small RNA. wt: non-transformed, wild type ‘Angeleno’; AG7303–96: PpeIFiso4G11-silenced, transgenic ‘Angeleno’ Japanese plants.

**Additional file 9: Table S3.** Differentially expressed miRNA species and their targets in wild-type ‘Angeleno’ and in *PpeIFiso4G11*-silenced transgenic line. ^1^ Treatment refers to the non-infected (NI) to infected, with either PPV-M or PPV-D, status of the plants. ^2^ Genotype refers either to wild-type, non-transformed plum (‘Angeleno’ NT) or to AG7303–96 transgenic plants (named here ‘*PpeIFiso4G11*-silenced’). Total normalized reads were calculated dividing the number of total reads of a specific molecule by the total number of reads in the library. Expectation for target genes were obtained from the pSRNA software [51]. The NCBI link allows to retrieve the identity and true/putative function of each target protein. A) The most differentially expressed miRNA species in *Prunus salicina* ‘Angeleno’ wild-type (non-transgenic) plants. B) The most differentially expressed miRNA species in *Prunus salicina* ‘Angeleno’ *PpeIFiso4G11*-silenced plants. C) miRNA species uniquely expressed either in *Prunus salicina* ‘Angeleno’ wild-type or *PpeIFiso4G11*-silenced plants.

**Additional file 10: Table S4.** List of primers used for the production of RNAi constructs, the verification of transgenic lines and the estimation of relative expression analysis. ^1^ In italics, sequences of the restriction sites used for cloning of the RNAi silencing constructs. ^2^ Fragment size without the restriction sites added in the primers.

## Data Availability

The datasets generated during the current study, i.e. small RNA NGS data (21 to 24 nt) are available at the INRA data portal (https://data.inra.fr/) where they can be freely retrieved under the link: https://data.inra.fr/dataset.xhtml?persistentId=doi:10.15454/O07SXQ.

## Abbreviations

eIF: Eukaryotic translation Initiation Factor
ELISA: Enzyme-Linked Immuno Assay
PPV: *Plum Pox Virus*
Q-RT-PCR: Quantitative Reverse Transcribed Polymerase Chain Reaction
