# Supplementary figures and images for: Silencing of one copy of the translation initiation factor eIFiso4G in Japanese plum (Prunus salicina) impacts susceptibility to Plum pox virus (PPV) and small RNA production
Source: BMC Plant Biol. 2019 Oct 22;19:440. doi: 10.1186/s12870-019-2047-9 (PMC6806492; doi:10.1186/s12870-019-2047-9)

Time point (ELISA) based observations

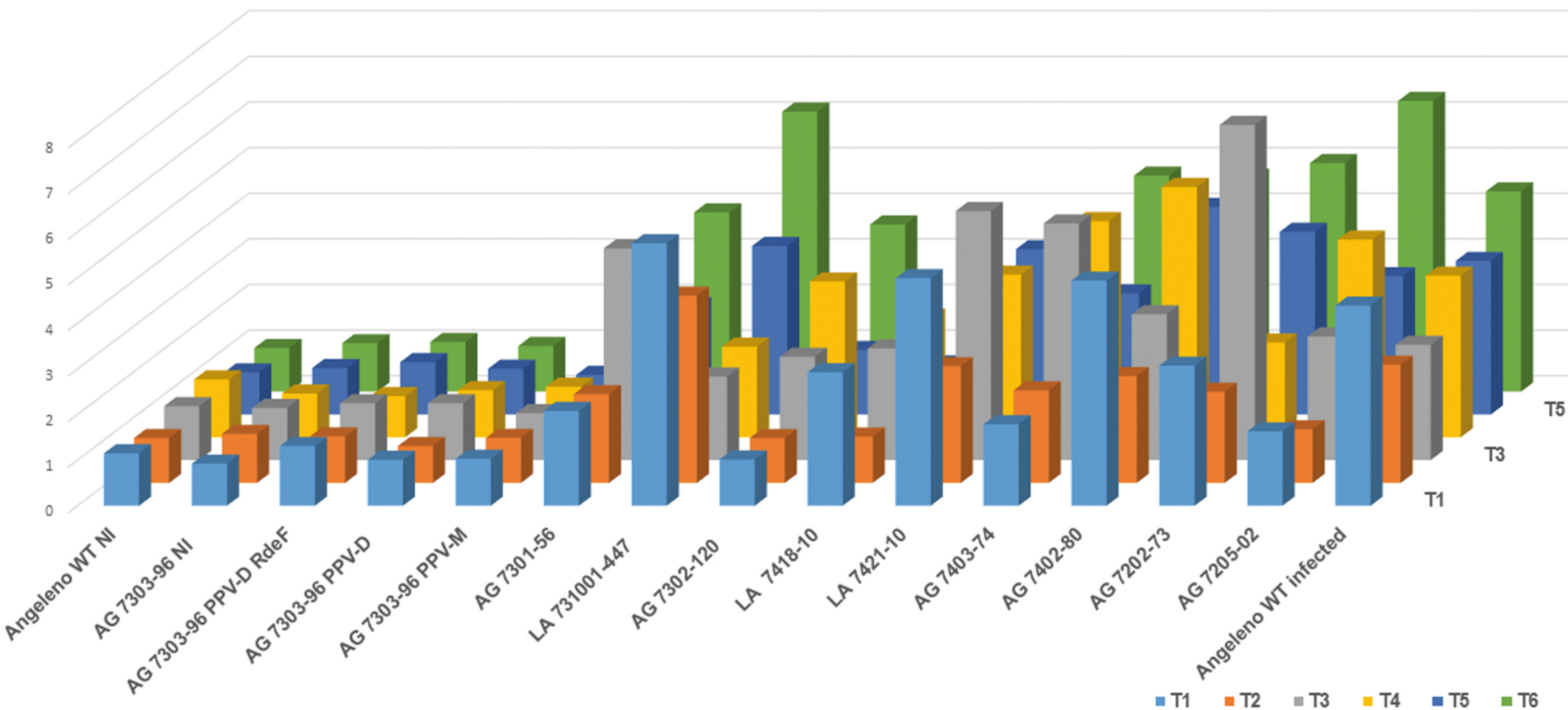

Supplement: Supplementary file 1 — Additional file 1: Figure S1. DAS-ELISA of wild type and transgenic Japanese plum lines following inoculation with Plum Pox Virus (PPV) over three vegetative cycles. Values represent the mean optical density values of three to four replicates per transgenic line tested for PPV infection over 3 vegetative cycles. AG: ‘Angeleno’ transgenic lines; LA: ‘Larry Ann’ transgenic lines. Numbers starting with 73 were transformed with pH 12-PpeIFiso4G11; 74 with pH 12-PpeIFiso4G10 and 72 with pH 12-PpeIF4G. All plants were grafted on rootstocks infected with PPV-M except NI (non-infected) and the plants noted PPV-D (PPV-D8 and PPV-D RdF Rouge de Fournés isolates). [file 12870_2019_2047_MOESM1_ESM.pdf]

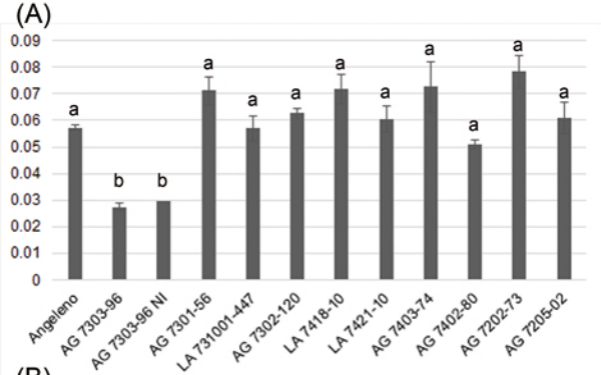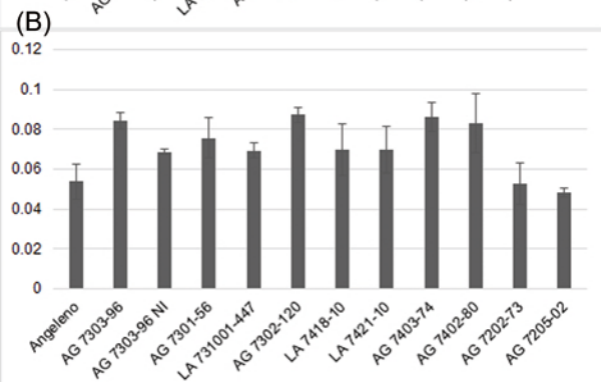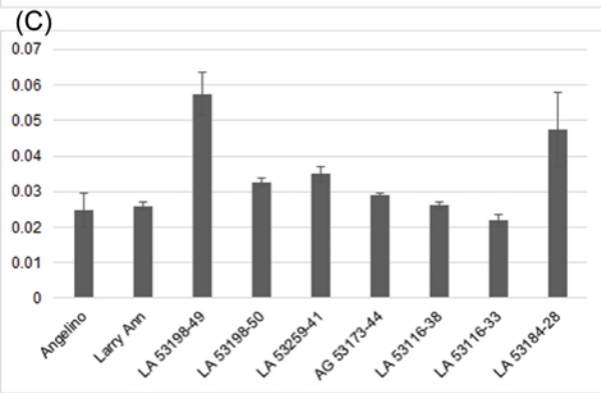

Supplement: Supplementary file 2 — Additional file 2: Figure S2. Relative expression analysis of PpeIFiso4G10 (A), PpeIF4G (B) and PpeIF4E (C) in leaves of transgenic Japanese plum trees. Transcript levels were analyzed by qRT-PCR. All values were normalized to the TEFII reference gene and then compared to the wild type ‘Angeleno’ gene expression level. Error bars represent the standard deviation of two biological replicates each analyzed in triplicate. The standard deviation between replicates is indicated by vertical lines. Statistical analysis was performed using the Kruskal–Wallis rank sum test in R software v. 3.2.5. Transgenic and wild type Japanese plum lines labelled with the same letter are statistically identical (P value < 0.05). No significantly different values of PpeIF4G (B) and PpeIF4E (C) expression was evidenced by the Kruskal-Wallis test at P value ≤0.05. AG: transgenic ‘Angeleno’ plum lines; LA: transgenic ‘Larry Ann’ plum lines. The transgenic line numbers are corresponding to the ones depicted in Fig. 1b. [file 12870_2019_2047_MOESM2_ESM.pdf]

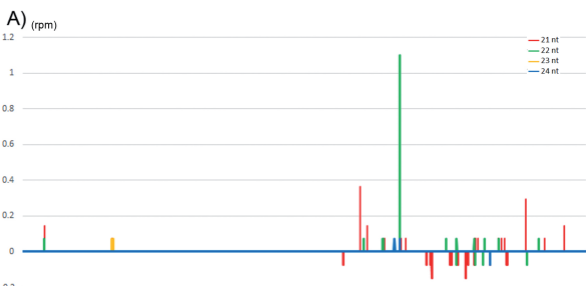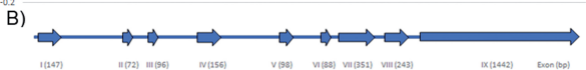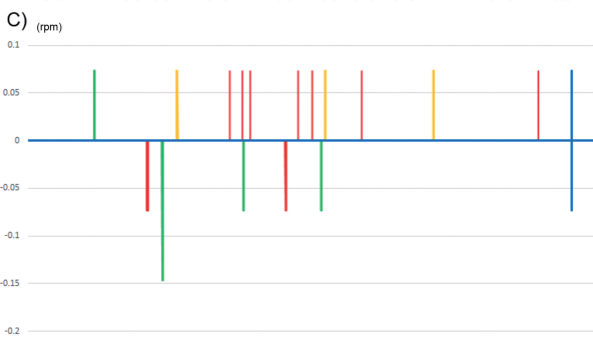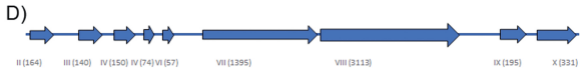

Supplement: Supplementary file 3 — Additional file 3: Figure S3. Accumulation of PpeIFiso4G10 (A) and PpeIF4G (C) specific siRNA in the AG7303–96 transgenic line. (B) is representing PpeIFiso4G10 and (D) PpeIF4G sequences. (rpm) reads per million with a total count of 85 and 20 reads over the PpeIFiso4G10 and PpeIF4G loci, respectively. [file 12870_2019_2047_MOESM3_ESM.pdf]

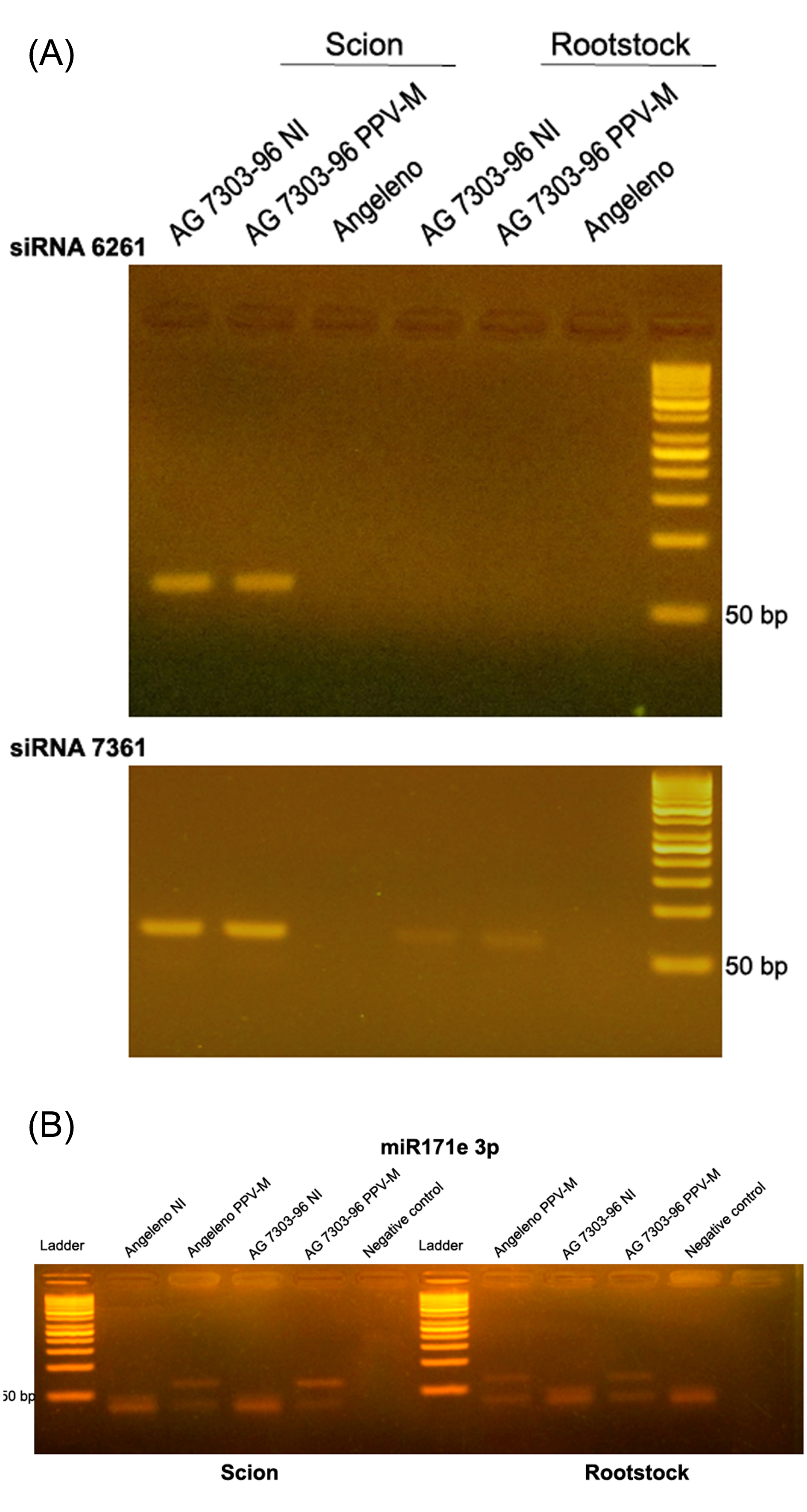

Supplement: Supplementary file 4 — Additional file 4: Figure S4. Stem-loop reverse transcription-polymerase chain reaction (RT-PCR) detection of selected siRNAs and miRNAs. (A) Transgene-derived siRNAs (#6261 and 7361, respectively) were detected in AG 7303–96 transgenic Japanese plums as well as in peach GF305 rootstock (for #7361 exclusively). (B) Expression pattern of miR171e 3p in scions and rootstocks of non-transformed ‘Angeleno’ and transgenic AG 7303–96 Japanese plum trees. [file 12870_2019_2047_MOESM4_ESM.tif]

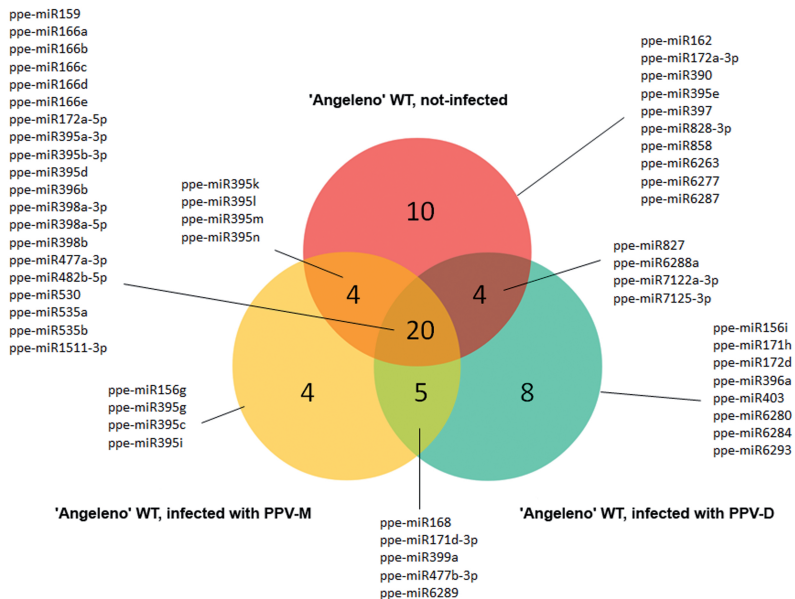

Supplement: Supplementary file 5 — Additional file 5: Figure S5. Venn diagram for the specific and shared miRNAs among the wild type, non-transgenic and AG7303–96 transgenic ‘Angeleno’ libraries. [file 12870_2019_2047_MOESM5_ESM.pdf]

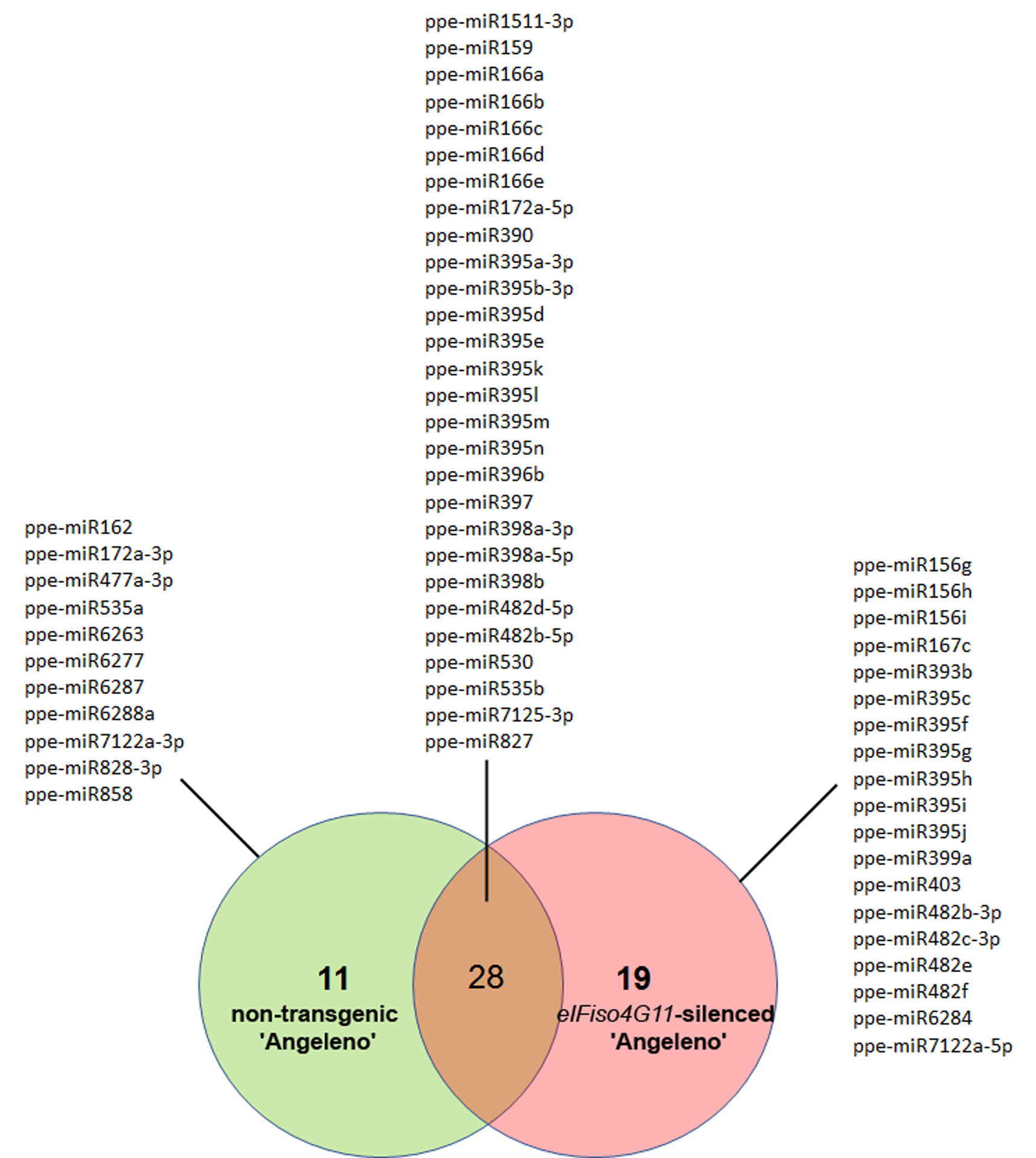

Supplement: Supplementary file 6 — Additional file 6: Figure S6. Venn diagram for the specific and shared miRNAs among the non-infected (NI), PPV-M or PPV-D infected wild type (non-transgenic) ‘Angeleno’ libraries. [file 12870_2019_2047_MOESM6_ESM.tif]
